# Contrast-induced acute kidney injury and nephrogenic systemic fibrosis in children

**DOI:** 10.1007/s00467-025-06916-w

**Published:** 2025-08-11

**Authors:** Alice Ming‑jie Chuah, Jonathan Chen, Alison Lap‑tak Ma, Kin Fen Kevin Fung, Eugene Yu‑hin Chan

**Affiliations:** 1 Paediatric Unit, Hospital Sultanah Bahiyah, Langgar, Malaysia; 2 Paediatric Nephrology Centre, Hong Kong Children’s Hospital, Kowloon, Hong Kong SAR; 3 Department of Radiology, Hong Kong Children’s Hospital, Kowloon, Hong Kong SAR; 4 Department of Diagnostic and Interventional Radiology, The Hospital for Sick Children, Toronto, Canada; 5 Department of Paediatrics, Faculty of Medicine, The Chinese University of Hong Kong, Shatin, Hong Kong SAR

**Keywords:** Contrast, Contrast-induced acute kidney injury, Gadolinium, Nephrogenic systemic fibrosis, Acute kidney injury, Children

## Abstract

Intravascular contrast media plays an important role in improving tissue and vascular characterisation in diagnostic imaging and image-guided intervention. Iodinated contrast media are commonly used in imaging modalities which utilise ionising radiation and gadolinium-based contrast agents (GBCA) in magnetic resonance imaging. Intravascular use of iodinated contrast medium is associated with contrast-induced acute kidney injury and GBCA with nephrogenic systemic fibrosis, both of which can lead to potentially significant adverse outcomes in patients with kidney impairment. However, most studies in the literature focus on adults and the evidence in the paediatric population is scarce. In this review, we aim to examine the consensus guidelines and studies in the paediatric population who require intravascular iodinated contrast media and GBCA. At the end, we propose an algorithm to approach the use of intravascular contrast media in children who need to undergo diagnostic imaging or image-guided intervention.

**Graphical abstract:**

**Figure:**
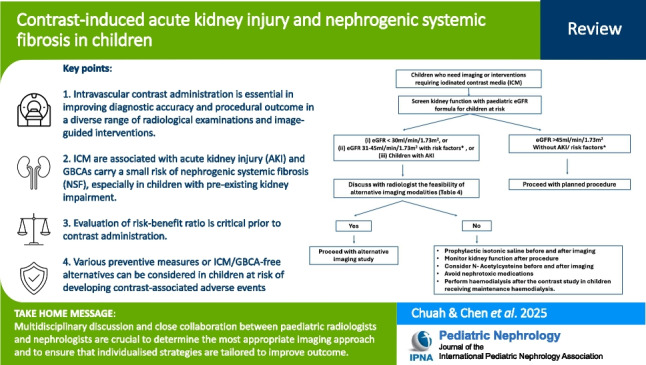
A higher resolution version of the Graphical abstract is available as Supplementary information

**Supplementary Information:**

The online version contains supplementary material available at 10.1007/s00467-025-06916-w.

## Introduction

Over the past decades, computed tomography (CT) and magnetic resonance imaging (MRI) have become essential clinical tools to establish disease diagnosis and evaluate treatment response. Intravenous administration of iodinated contrast media (ICM) for CT and gadolinium-based contrast agents (GBCAs) for MRI enhances soft tissue characterisation, delineates vascular structures, and increases diagnostic confidence [1, 2]. However, the use of contrast media carries potential risks in patients with kidney disease. Specifically, ICM has been associated with acute kidney injury (AKI), while GBCAs carry a risk—albeit low with current agents—of nephrogenic systemic fibrosis (NSF). Conversely, deferring or omitting contrast-enhanced imaging may result in delayed diagnosis or intervention, potentially compromising clinical outcomes [3].

Although there are published guidelines pertaining to the use of contrast media in the adult population [1, 2, 4], most recommendations are based on adult data, with limited evidence specific to children with kidney disease. Consequently, such guidance may not be directly applicable to paediatric patients. In this review, we aim to summarise the current evidence on contrast-related AKI and NSF in children and adults through case-based discussion. We will attempt to develop a pragmatic approach to optimise the safe and effective use of contrast agents among children with kidney disease.

## Case presentation (case 1)

A 5-year-old boy with acute lymphoblastic leukaemia, receiving intensive chemotherapy, was admitted for high-grade fever and diffuse abdominal pain. Physical examination revealed diffuse tenderness, guarding, and rebound tenderness. C-reactive protein was elevated. His estimated glomerular filtration rate (eGFR) was 55 ml/min/1.73 m^2^. He was given systemic antibiotics for neutropenic fever and systemic sepsis. The attending surgeon suggested a contrast-enhanced CT to delineate the cause of abdominal pain. What is the risk of developing contrast-induced AKI (CI-AKI) and how can we prevent it?

### Use of intravascular iodinated contrast media in radiological examinations

ICM are commonly administered intra-arterially or intravenously in digital subtraction angiography or CT to visualise vascular structures or improve characterisation of soft tissue lesions [4, 5]. Iodine plays a key role in attenuating the X-ray through the photoelectric effect; therefore, it allows soft tissue with increased absorption of ICM to be more distinct on imaging modalities involving ionising radiation [6]. The following discussion focuses on water-soluble iodinated contrast media. We do not cover ethiodised poppy seed oil, which is used for lymphangiography, trans-arterial chemoembolisation, or sclerotherapy [7].

ICM can be broadly categorised based on chemical structure into ionic and non-ionic agents [6]. Due to their higher toxicity profiles, ionic ICM are now rarely used for intravascular applications. Another critical characteristic of ICM is osmolality. Most modern intravascular ICMs in clinical use are either low-osmolar contrast media (LOCM) or iso-osmolar contrast media (IOCM). LOCM typically have an osmolality of around 500–800 mOsm/kg, which is approximately two to three times that of both IOCM (~ 290 mOsm/kg) and human serum (~ 290 mOsm/kg). High-osmolar contrast media (HOCM), with an osmolality of around 1400 mOsm/kg, are associated with a greater risk of adverse effects and are now largely obsolete in intravascular imaging [6].

All ICM exhibit low protein binding and undergo rapid distribution from the intravascular compartment to well-perfused organs such as the brain, liver, and kidneys. In contrast, distribution to poorly perfused tissues such as fat and bone occurs more slowly. Nonetheless, redistribution half-lives are typically rapid—ranging from 2 to 5 minutes for most agents [8]. Importantly, all currently available ICM are eliminated unchanged via glomerular filtration, with no significant metabolism or tubular reabsorption [6]. In individuals with normal kidney function, the elimination half-life of ICM is generally between 1.8 and 2.3 hours [9]. Based on this pharmacokinetic profile, a minimum interval of 12 hours between successive contrast-enhanced CT scans is recommended to ensure near-complete elimination of the contrast agent [9]. In urgent clinical scenarios, a shorter interval of at least 4 hours may be considered [9]. In patients with kidney impairment, however, contrast elimination is markedly delayed, with reported half-lives of up to 27 hours [9]. As such, near-complete clearance may require up to 7 days, while approximately 75% of the contrast load is cleared within 2.5 days. This may serve as a practical minimum interval before repeating contrast-enhanced imaging in this population.

### Nomenclature and definition

CI-AKI is different from contrast-associated AKI (CA-AKI). CA-AKI is defined as the development of AKI, of any degree, within 48 hours following ICM exposure [4]. There is no direct causal relationship between contrast exposure and AKI. In the presented case with acute leukaemia, multiple factors may have contributed to the development of AKI, including systemic sepsis and nephrotoxic antibiotics [10]. In contrast, in CI-AKI, there is an established causal relationship between contrast exposure and AKI, and other potential contributing factors are ruled out. Several international organisations, including American College of Radiologists (USA), Royal College of Radiologists (UK), and Canadian Association of Radiologists, define the staging/severity of AKI in accordance with the Kidney Disease: Improving Global Outcomes (KDIGO) guidelines [4, 11–13].

### Pathophysiology of CI-AKI

The exact pathogenesis of CI-AKI is not well understood. It is likely a result of direct tubular toxicity imposed by the ICM and impairment of intra-renal haemodynamics due to vasoconstriction of peritubular capillaries and glomerular capillaries [14–16]. A combination of direct tubular epithelial cell toxicity and endothelial cell dysfunction leads to tubular necrosis, leading to an increased synthesis of pro-inflammatory cytokines and reactive oxygen species—ultimately perpetuating a vicious cycle [17]. The physicochemical properties of ICM, particularly high osmolality, viscosity, and injected volume, exacerbate this nephrotoxic potential [16]. As a result of these adverse effects, the intravascular use of high-osmolar ICM has been largely abandoned [18]. Their use is now mostly confined to gastrointestinal fluoroscopic studies and cystourethrography [18, 19].

### Incidence and risk factors for developing contrast-induced acute kidney injury

A recent systematic review and meta-analysis evaluated a total of 120 studies on CI-AKI in the literature [20]. The overall incidence of CI-AKI and AKI necessitating acute kidney support were 9.06% (95% CI, 8.53–9.58%) and 0.52% (95% CI, 0.37–0.70%), respectively. Considerable variation in the epidemiology was observed across different study designs. The incidence of CI-AKI could range from 15.0% in clinical trials to 7.9% in retrospective studies, suggesting differences in monitoring policies and AKI definitions used. The risk of AKI was substantially higher amongst patients receiving intra-arterial contrast administration compared to intravenous contrast media (9.6% vs. 2.6%). Furthermore, a higher incidence was also observed among heart-related procedures (9.9% vs. 5.4%). This may be attributable to the presence of concurrent risk factors associated with underlying cardiac conditions, such as congestive heart failure, diabetes mellitus, impaired cardiac function, and chronic hypertension.

There is conflicting evidence in the adult literature regarding the risk of CI-AKI following intra-arterial versus intravenous administration of ICM. A randomised trial showed a higher incidence of AKI following cardiac catheterisation compared to CT angiography, particularly in patients undergoing cineventriculography or those with pre-existing chronic kidney disease (relative risk 2.4; 95% CI, 1.1–5.0; *p* = 0.02) [21]. However, the independent contribution of intra-arterial ICM administration to nephrotoxicity remains difficult to quantify due to confounding factors, including the risk of thromboembolic events from catheter manipulation and the abrupt, concentrated delivery of contrast directly to kidneys [22].

One of the established risk factors is pre-existing kidney impairment, where the current evidence suggests a threshold cutoff at < 30 ml/min/1.73 m^2^ [4]. Four large-scale adult studies showed that ICM was not a risk factor for developing CI-AKI in patients with an eGFR > 45 ml/min/1.73 m^2^ [23–26]. The occurrence of CI-AKI was also rare among those with an eGFR between 30 and 44 ml/min/1.73 m^2^ [23–26]. The association of kidney function and CI-AKI was more conflicting in CKD stages 4 and 5 [23–26]. Since it is challenging to differentiate CI-AKI from CA-AKI clinically, the absolute risk of developing CI-AKI remains unclear in patients with pre-existing kidney impairment. Patients at high risk of developing AKI might have been excluded from contrast exposure, which may further confound the data. On the other hand, more paediatric data is required to assess the risk of developing CI-AKI in children with an eGFR of 30 ml/min/1.73 m^2^ or above.

The estimation of GFR based on serum creatinine alone may be inaccurate, particularly in young children or children with low muscle mass, where additional measurement of cystatin C may provide a more accurate assessment of kidney function. In the setting of concomitant AKI, serum creatinine is unreliable, and alternative biomarkers such as neutrophil gelatinase-associated lipocalin (NGAL) in blood and/or urine have been utilised for early detection of CA-AKI in some centres [27]. It should be noted that there is limited data that suggests contrast exposure may worsen or prolong AKI [4]. However, as these patients are susceptible to kidney injuries, contrast use should ideally be avoided whenever possible [4]. Other risk factors of CI-AKI, including advanced age and dehydration, have been described in the adult population, and relevant factors for the paediatric population are summarised in Table 1 [28–30].

**Table 1:** Risk factors associated with contrast-induced acute kidney injury in children [11]. Double asterisks (**) denote reported association in the paediatric population

**Risk factors**
Diabetes mellitus Pre-existing kidney impairment** Acquired kidney disease** Increased body mass index** Nephrotoxic antibiotics** (e.g. aminoglycoside) Nephrotoxic medications (angiotensin-converting enzyme inhibitors, diuretics, non-steroidal anti-inflammatory drugs, chemotherapy such as cisplatin) Reduced effective circulatory volume (e.g. hypotension, dehydration, hypoalbuminaemia) Impaired cardiac function (e.g. congestive heart failure, low left ventricular ejection fraction, acute myocardial infarction, cardiogenic shock) Kidney transplant Intra-arterial administration of iodinated contrast media

In children, data pertaining to CI-AKI are scarce and are limited by the retrospective nature of the reporting studies. In a retrospective observational study by Gilligan et al., 925 hospitalised children who underwent intravenous contrast media were matched and compared to 925 patients who had ultrasonography [31]. The incidence of AKI was similar between the contrast-enhanced CT and ultrasonography groups (2.4% vs. 2.6%). Sensitivity analyses among patients with an eGFR ≥ or < 60 ml/min/1.73 m^2^ also showed comparable rates of AKI. Thus, the development of AKI was not associated with intravenous ICM and might be attributed to other risk factors including a lower baseline eGFR, a high body mass index, concurrent acquired kidney disease, and an increasing number of nephrotoxic antibiotics. Notably, better kidney function reduces the risk of developing CI-AKI (odds ratio 0.99, 95% CI 0.98–0.995, *p* = 0.001).

Another multicentre French study reported an incidence of CI-AKI of 10.3% among 346 paediatric subjects undergoing contrast-enhanced CT [32]. Children with CI-AKI, compared to those without, were associated with higher rates of 30-day hospital readmission (25% vs. 8.6%, *p* = 0.02) and hospital mortality (16.7% vs. 6.7%, *p* = 0.09). No specific risk factor for developing CI-AKI was identified. The incidence of CI-AKI observed in this study might be over-reported, since CA-AKI could not be confidently differentiated from CI-AKI due to the retrospective study design and lack of patient controls who were not exposed to ICM.

A large retrospective single centre including 10,407 children and adolescents which evaluated the risk of AKI following intravenous ICM administration for CT examination concluded that the incidence of AKI after contrast-enhanced CT was very low (1.4% (123/8844)) and comparable with the non-contrast CT group (1.6% (171/10,533); *p* = 0.18). Consistent with previous studies, the incidence of AKI was significantly higher in patients with an eGFR < 60 ml/min/1.73 m^2^ who underwent contrast-enhanced CT (8.5% (10/118)) compared to those who underwent non-contrast CT (2.7% (169/6238); *p* < 0.001). While type II error was likely minimised given the large cohort size, it was difficult to eliminate potential confounders and pre-selection bias in the study.

### Preventive measures

Every effort should be made to prevent CI-AKI, since this may lead to cumulative injuries in children with CKD. Baseline kidney function should be evaluated to identify at-risk patients. In adults, this includes a personal history of kidney disease (known CKD, history of AKI, dialysis, proteinuria, and prior kidney surgery or ablation), diabetes mellitus, and use of metformin or metformin-containing medications [4]. While specific guidance for paediatric patients is not available, relevant risk factors such as extreme prematurity or low birth weight should be considered when determining the need for screening. The use of ICM should be avoided in high-risk patients with eGFR < 30 ml/min/1.73 m^2^. However, this may not be feasible, and therefore a joint discussion between paediatric radiologists and nephrologists is crucial to decide on the most appropriate imaging approach. Alternative non-contrast imaging may be considered, including non-contrast CT, MRI, and ultrasonography. When ICM is indicated and cannot be avoided, preventive measures should be employed as discussed in the following sections. Early detection of CI-AKI is also important. For this purpose, we would monitor kidney function 12 and 72 hours following ICM exposure in at-risk patients with impaired kidney function and aforementioned risk factors.

## The choice and dosage of iodinated contrast medium

The osmolality of ICM has been implicated in the pathogenesis of contrast-induced nephrotoxicity. A meta-analysis has demonstrated that the incidence of AKI is higher with HOCM compared to LOCM in patients with pre-existing renal impairment [33]. Regarding the comparison between LOCM and IOCM, current adult data does not support a clear clinical advantage of one over the other in terms of nephrotoxicity with regard to intravenous administration. Several studies have failed to demonstrate a significant benefit of iodixanol, the most widely used IOCM, over LOCM in preventing CA-AKI or CI-AKI. This finding is supported by a meta-analysis of 25 randomised controlled trials, which found no significant difference in the incidence of CA-AKI between iodixanol and LOCM when administered intravenously [34]. However, in patients with intra-arterial administration or renal insufficiency, iodixanol was associated with a reduced risk of CA-AKI [34].

In the context of the paediatric population, similar findings were shown in a multicentre randomised controlled trial comparing intravenous iso- vs. non-osmolar ICMs for contrast-enhanced CT examinations [35]. No significant difference in incidence of CA-AKI was found in children with normal kidney function. Given HOCM is rarely used intravascularly in modern clinical practice, there is likely little clinical implication for choosing between LOCM or IOCM in the context of intravenous administration.

Currently, even in patients with high risk of CI-AKI, on-label standard dosing is recommended for imaging studies requiring intravenous ICM administration if the benefit outweighs the risk. Dose reduction is generally not recommended as this may result in suboptimal or non-diagnostic images [4]. However, in the context of intra-arterial ICM administration, where a dose-dependent relationship with nephrotoxicity has been observed [36], it is prudent to adhere to weight-based dose limits—typically 4–5 ml/kg for neonates and 6–8 ml/kg for infants and young children [37]. Additional strategies to optimise contrast usage, while minimising unnecessary exposure and wastage, include aspiration of residual ICM within the catheter “dead space” and contrast dilution techniques, commonly at a 1:1 ratio with normal saline.

### Volume expansion

Volume expansion is the most evidence-based practice to prevent CI-AKI [38]. The incidence of CI-AKI was significantly lower (20.4% vs. 35.2%) in patients receiving prophylactic volume expansion for percutaneous coronary intervention [39]. In particular, the administration of isotonic saline before and after ICM administration is a protective factor against CI-AKI [40, 41]. Volume expansion prevents dehydration, and it also reduces cellular damage at the medullary tubular segments through increased renal blood flow, glomerular filtration, increased volume of urine flow, and dilution of the contrast medium [38, 40–42]. Volume expansion is thus recommended in the guidelines published by KDIGO and the American College of Radiology (ACR) [4, 11]. While volume expansion is not required in the general adult population with eGFR > 30 ml/min/1.73 m^2^, patients with eGFR between 30 and 44 ml/min/1.73 m^2^ can be offered at discretion in the presence of additional risk factors and/or recent AKI [4]. In our practice, we consider volume expansion in children with eGFR < 45 ml/min/1.73 m^2^ since paediatric CKD is uncommon and these patients often maintain adequate urine output.

Isotonic crystalloid is the choice of fluid for volume expansion. While 0.9% normal saline (NS) is the most commonly prescribed fluid, solutions containing lower concentrations of chloride such as Plasma-Lyte may be more physiological but require further investigations. The optimal timing, infusion rate, and total volume remain unclear. In the ACR guidelines, volume expansion starts 1 hour prior to the contrast study and continues until 3 to 12 hours after contrast exposure [4]. Longer duration of volume expansion (12 hours) has been associated with a lower risk of CI-AKI when compared to shorter regimens. The prescription is either by body weight (1–3 ml/kg/h) or flat rate (500 ml before and after examination). However, this dosing based on body weight may account for only 10–30% of the daily fluid requirement (100 ml/kg/day) in young children. In our clinical practice, we administer fluid at an infusion rate (per hour) of 100–150% of the standard maintenance requirement to achieve effective volume expansion, with adjustment based on urine output to prevent fluid overload among patients with pre-existing oliguria. Liberal oral fluid and solute intake complement intravenous fluid administration [43]; however, its role as an independent preventive measure is yet to be investigated in future studies [41].

### Sodium bicarbonate and N-acetylcysteine

Earlier studies demonstrated that the use of sodium bicarbonate reduced the risk of developing CI-AKI [44, 45]. The roles of sodium bicarbonate and *N*-acetylcysteine in preventing CI-AKI were further assessed in a large randomised controlled trial, the PRESERVE study, in patients undergoing angiography [46]. A total of 5177 high-risk patients were randomised to receive intravenous 1.26% sodium bicarbonate versus 0.9% NS and 5 days of oral *N*-acetylcysteine versus oral placebo. The primary outcome was a composite outcome comprising death, dialysis initiation, or a rise in serum creatinine levels > 50% from baseline at 90 days. CA-AKI was assessed as the secondary outcome. A similar proportion of patients receiving bicarbonate or 0.9% NS developed the composite outcome and CA-AKI (4.4% vs. 4.7%, respectively). Consequently, bicarbonate and 0.9% NS were considered equally effective in preventing CI-AKI, and the ACR prefers 0.9% NS to avoid additional pharmacist compounding [4].

Despite a lack of evidence, the use of *N*-acetylcysteine in conjunction with isotonic volume expansion had been suggested for patients at risk of CI-AKI [11]. In the aforementioned PRESERVE trial, the use of *N*-acetylcysteine did not impact on the development of CI-AKI (*N*-acetylcysteine vs. placebo, 4.6% vs. 4.5%). The medication is therefore no longer recommended in adults [4]. Since high-quality evidence is not available in paediatric patients, we still prescribe *N*-acetylcysteine to children at risk of CI-AKI due to a relatively low cost and safe drug profile. *N*-Acetylcysteine is often given at 600 mg twice daily 24 hours before and after the procedure in adults [47]. The dosing for paediatric patients is less established. In our practice, we prescribe the adult dosing for older children and adolescents, and a lower dose at 300 mg twice daily before and after the procedure in younger children.

### Withholding nephrotoxic drugs

Patients with CKD are often prescribed multiple medications, including renin–angiotensin–aldosterone system inhibitors (RAASi) to slow kidney progression [48]. Some of these medications are potentially nephrotoxic, including non-selective non-steroidal anti-inflammatory drugs and antimicrobials. If feasible, non-essential nephrotoxic medications should be withheld 24–48 hours before planned ICM administration [49]. However, withholding RAASi prior to ICM administration remains controversial as discontinuation did not prevent the development of CI-AKI in a randomised trial involving 220 adult patients [50].

### Other non-beneficial preventive measures

Prophylactic dialysis is not indicated in patients with CKD stages 4–5 who are not on maintenance dialysis, owing to the lack of clinical benefit and potential complications and cost [4]. In a systemic review by Cruz et al. which evaluated 11 clinical trials on prophylactic dialysis with 1010 patients [51], the incidence of CI-AKI was not different in the dialysis and non-dialysis groups (23.3% vs. 21.2%).

The use of mannitol or frusemide to induce diuresis also failed to prevent CI-AKI. In a historical trial, 78 patients with CKD undergoing cardiac angiography were randomised to receive either hypotonic solution (0.45% saline), NS plus frusemide, and NS plus mannitol [52]. A significantly higher proportion of patients receiving mannitol (28%) or frusemide (40%) developed CI-AKI, compared to the saline group (11%).

### Special considerations in the dialysis population

In contrast to adults, children with kidney failure due to congenital anomalies and cystic kidney disease often have significant residual urine. Consequently, it is important to prevent CI-AKI in order to preserve residual kidney function. Patients who are not anuric should be treated similarly with volume expansion, with or without *N*-acetylcysteine. The volume prescribed should be adjusted to avoid fluid overload. Furthermore, ICM can be efficiently removed by haemodialysis or haemodiafiltration. While acute or additional dialysis or continuous kidney replacement therapy should not be initiated solely because of an ICM exposure [4], the dialysis schedule of patients undergoing maintenance haemodialysis can be synchronised with the procedure to reduce the impact of contrast exposure, without additional risk and cost [53]. Peritoneal dialysis, which is performed daily in most cases, also eliminates ICM but at a slower rate compared to haemodialysis [54].

## Management of case 1

After discussion with the radiologist, the patient proceeded with contrast-enhanced CT with iso-osmolar contrast. He was given volume expansion with NS before and after the contrast study and was additionally prescribed *N*-acetylcysteine. He did not receive any frusemide or mannitol. The CT suggested a diagnosis of neutropenic colitis, and the patient responded to systemic antibiotics and granulocyte colony-stimulating factors (G-CSF) and other supportive therapies. Kidney function was monitored after the contrast study and did not show any evidence of CI-AKI.

### Case presentation (case 2)

A 12-year-old girl with a background of autoimmune disease and receiving peritoneal dialysis came in via emergency for pyrexia and encephalopathy. Neurologists suggested urgent gadolinium-enhanced MRI brain for further work up. Can gadolinium be used in a dialysis patient? What is the risk of developing NSF?

### Nephrogenic systemic fibrosis

NSF is a debilitating and potentially fatal condition. It is generally accepted that an exposure to gadolinium-based contrast agents (GBCAs) is a pre-requisite to develop NSF [55–57]. While the pathophysiology remains unclear, kidney impairment prolongs the physiologic half-life of GBCAs and increases the circulating concentrations of the endogenous metals iron, copper, and zinc. Both effects promote the dissociation of gadolinium cation (Gd^3+^) from the chelating ligands. Once displaced, free gadolinium cation combines with endogenous ions such as phosphate to form complexes that precipitate in tissues and elicit a fibrotic process, leading to the clinical manifestations of NSF [57]. This means that NSF occurs almost exclusively in patients with kidney impairment either in the context of severe AKI or advanced CKD [2, 58].

Clinical manifestations include skin thickening, contractures, pruritus, hyperpigmentation, and scleral plaques [59]. The most debilitating feature is fibrosis of internal organs, including lung, oesophagus, and heart [60]. The diagnosis is established through clinical features and confirmed upon skin biopsy [61].

### Gadolinium-based contrast agents

GBCAs are the most commonly used contrast media for MRI examinations and have been first approved by the US Food and Drug Administration (FDA) since 1988 [2]. The utility of GBCAs capitalises on the paramagnetic properties of the gadolinium ion, which possesses seven unpaired electrons. This results in the shortening of the T1 spin–lattice relaxation time and increased signal intensity on T1-weighted images, thereby enhancing diagnostic information, particularly in vascular imaging and soft tissue characterisation [62]. Various organic ligands are used to chelate the toxic gadolinium cation in GBCAs, preventing the release of free gadolinium into the bloodstream, thereby improving the stability and safety profile of the contrast agent [63]. Structurally, GBCAs can be classified into two main groups based on their ligand structures, namely linear and macrocyclic GBCAs. The ACR, FDA, and European Medicines Agency (EMA) classify GBCAs according to the risk of developing NSF. The classification of GBCAs by the ACR manual is summarised in Table 2 [4]. Group I GBCAs are all linear GBCAs, while group II GBCAs are mostly macrocyclic GBCAs with the exception of gadobenate dimeglumine (MultiHance).

**Table 2:** Classification of GBCAs according to the risk of developing NSF

Group of GBCAs	Risk of NSF	Examples
Group I	High	Gadodiamide (Omniscan), gadopentetate dimeglumine (Magnevist), and gadoversetamide (OptiMARK)
Group II	Few unconfounded cases of NSF	Gadobenate dimeglumine (MultiHance), gadobutrol (Gadavist), gadoteric acid (Dotarem), gadoteridol, and gadopiclenol
Group III	Data remains limited regarding the risk of NSF, but only few cases have been reported	Gadoxetate disodium (Eovist)

### Epidemiology of NSF and associated factors

The reported incidence of NSF is highly heterogeneous [64]. In a systematic review comprising 370 biopsy-proven paediatric and adult cases of NSF, the incidence could range from 0 to 18% [64]. The peak occurrence of NSF was between 51 and 60 years of age. Importantly, 99% of the confirmed cases had an exposure to group I GBCAs. Second, up to 80% of subjects with NSF received maintenance dialysis. These findings indicate that patients with advanced CKD stages 4–5 and kidney failure on dialysis are at the highest risk of developing NSF. Group 1 GBCAs should not be administered in such groups of patients. The availability of newer generations of GBCAs has substantially reduced the risk. Woolen et al. reviewed the incidence of NSF from 16 studies involving 4391 patients with CKD stages 4–5 who received group II GBCAs [65]. None of the patients developed NSF, and the pooled incidence of developing NSF was 0%, with an upper bound of 95% confidence interval being 0.07%.

Another attributive factor is the GBCAs’ dosing. High-dose GBCA above standard dosing is associated with an increased rate of NSF [66]. In a large bi-centre study, none of the 74,124 patients receiving the standard dose of GBCAs developed NSF [43]. In contrast, 15 of the 8997 patients who received a higher dose regimen were confirmed to have NSF (0 vs. 0.17%; *p* < 0.01) [67]. Therefore, GBCAs should be given at standard dosing unless a higher dose is clinically justified.

To date, there is only limited data on NSF in the paediatric population. In a systematic search within the US FDA’s Adverse Event Reporting System and the International Center for Nephrogenic Systemic Fibrosis Research, only 23 children were reported to develop NSF between 1997 and 2012 [68]. Of these patients, 17 (73.9%) had a documented exposure to GBCAs, where 12 subjects received group 1 GBCAs, and the type of GBCAs prescribed was not documented in the remaining five patients. Eight children were on chronic dialysis (group 1 GBCAs, *n* = 5; unknown GBCAs, *n* = 3), and two subjects had severe AKI necessitating acute kidney support (both received group 1 GBCAs). Six patients had CKD. It thus concurs with adult data that group 1 GBCAs and kidney failure are the most important risk factors for developing NSF in children. Interestingly, no cases of NSF have been reported in neonates or infants, despite their physiologically immature renal function and estimated glomerular filtration rate (eGFR) values frequently falling below 30 ml/min/1.73 m^2^ [69].

### Preventive measures on NSF

Although the incidence of NSF with group II GBCAs is considered extremely low, valid concerns remain for patients with severe kidney impairment. In this context, careful identification of at-risk individuals, as outlined earlier, remains crucial. When GBCA-enhanced MRI is deemed clinically necessary, the lowest diagnostic dose should be used.

According to the US FDA, three group I GBCAs—gadopentetate dimeglumine (Magnevist), gadodiamide (Omniscan), and gadoversetamide (OptiMARK)—are contraindicated in patients with AKI or stage 4–5 CKD [70]. A Joint Consensus Statement from the ACR and the National Kidney Foundation concluded that the risk of NSF following group II GBCA administration is extremely low and that the potential harms of delaying or withholding a clinically indicated group II GBCA-enhanced MRI in patients with AKI or eGFR < 30 ml/min/1.73 m^2^ may outweigh the risk of NSF [2].

**Fig. 1:**
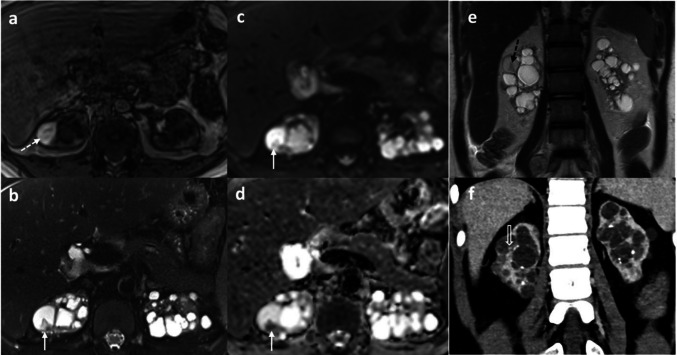
Plain MR abdomen screening in a 15-year-old boy with acquired cystic kidney disease on long-term peritoneal dialysis. **a**–**d** Axial plain MR abdomen shows one of the cysts has high signal (dashed white arrow) on T1-weighted sequence (**a**), and an intracystic nodule with low signal on T2-weighted sequence (**b**) and restricted diffusion (**c**, **d**), suspicious of malignant transformation. **e** Coronal plain MR abdomen shows the same intracystic nodule. Iodinated contrast was used instead of gadolinium-based contrast agent to further evaluate the lesion. **f** Nodular enhancement within the cyst was observed on contrast-enhanced CT. Pathology revealed papillary adenoma

It is therefore imperative to ensure that GBCA administration is clinically justified, especially in patients who undergo repeated imaging. For example, in patients with kidney failure and acquired cystic kidney disease, non-contrast MRI can be a viable alternative for characterising complex renal cysts when serial follow-up scans are anticipated (Fig. 1) [71–74]. Multidisciplinary discussion with radiologists is essential in such cases to determine the appropriateness of GBCA use or to consider alternative imaging approaches—including non-contrast MRI sequences, alternative contrast agents, or other imaging modalities.

Finally, haemodialysis eliminates GBCAs and is more effective than peritoneal dialysis [75]. There is no randomised trial which has evaluated the efficacy of dialysis in preventing NSF. For patients on maintenance haemodialysis, the dialysis should be scheduled soon after GBCA exposure. For patients on peritoneal dialysis and those who are not receiving kidney replacement therapy, there is insufficient evidence to recommend prophylactic haemodialysis to prevent the development of NSF [2].

## Management of case 2

After detailed discussion between parents, radiologist, nephrologist and neurologist, it was decided that an urgent gadolinium-enhanced MRI scan would be required for diagnosis. MRI brain with standard dose of group II GBCAs was performed uneventfully and the diagnosis of cerebral vasculitis was made. The patient recovered with timely use of immunosuppression and did not develop NSF after the imaging.

### ICM- and GBCA-free alternatives

**Fig. 2:**
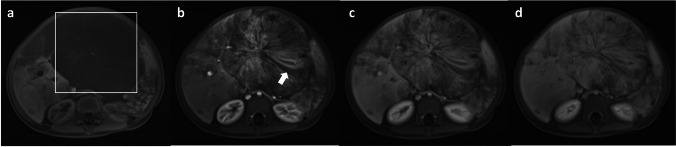
Magnetic resonance (MR) images of a 2-year-old girl with biopsy-proven hepatic angiosarcoma. **a** Unenhanced axial MR T1-weighted (T1W) scan showing a large left lobe mass with homogeneous intermediate T1W signal, limiting characterisation. **b**–**d** On the axial post-gadolinium contrast-enhanced arterial phase (**b**), portal venous phase (**c**), and delayed phase (**d**) MRI of the liver, the left lobe mass shows a progressive centrifugal and variegated enhancement pattern, with hypoenhancing striated areas (arrow in **b**), which possibly represent fibrous septations. Such enhancement pattern is highly suggestive of angiosarcoma. The white box in **a** demarcates the area imaged in the contrast-enhanced ultrasound in Fig. 3. Reprint with permission: [85]

**Fig. 3:**
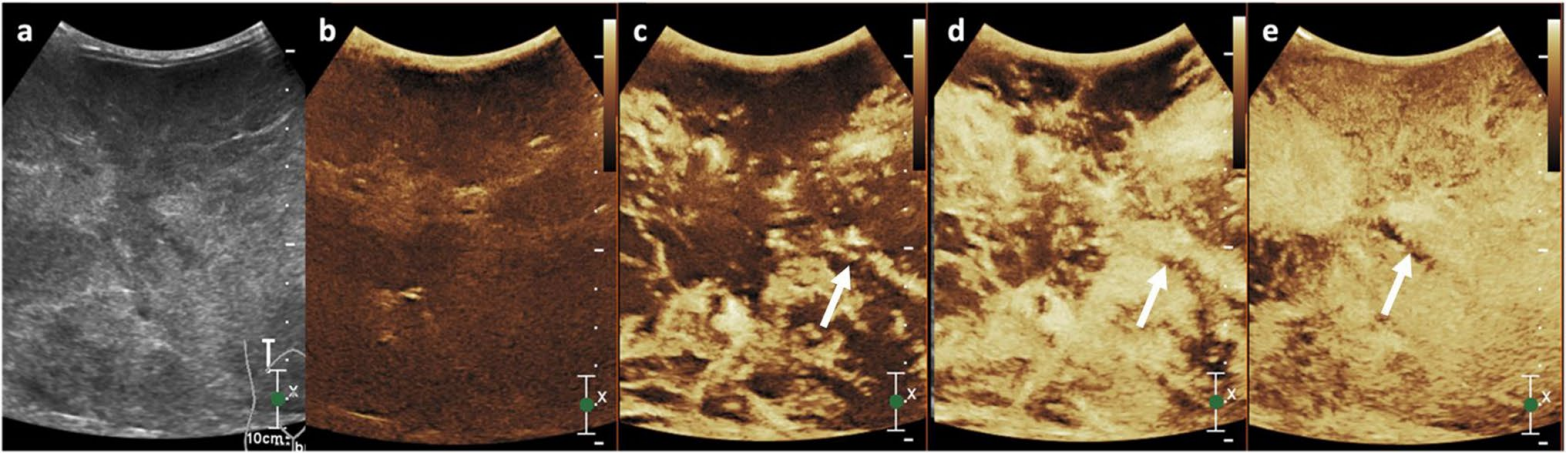
Follow-up contrast-enhanced ultrasound of the same 2-year-old girl, who developed acute kidney injury after chemotherapy treatment of the hepatic angiosarcoma. **a** Transverse greyscale (i.e. non-contrast-enhanced) ultrasound showed a large predominantly hypoechoic left lobe hepatic mass. **b**–**e** Transverse contrast-enhanced ultrasound in contrast-only mode in the arterial phase of the same mass at 5 s (**b**), 10 s (**c**), and 15 s (**d**), and in the portal venous phase 60 s after injection of the ultrasound contrast agent (**e**), shows the same progressive variegated multi-nodular enhancement seen on MR scan in Fig. 2. Similar hypoenhancing striations (white arrow) were observed, suggesting fibrous septation. Reprint with permission: [85]

Table 3 summarises advanced MR imaging modalities and novel contrast agents that may be considered in lieu of ICM and GBCAs, depending on the clinical situation and diagnostic requirements. In general, ultrasound is an excellent radiation-free imaging modality for young children, as their smaller body habitus results in minimal attenuation of the ultrasound beam, enabling high-resolution visualisation of various organs. The adjunctive use of non-nephrotoxic ultrasound contrast agents (UCA) has proven valuable in the characterisation of focal liver lesions (Figs. 2 and 3) and in the assessment of blunt abdominal trauma [76, 77]. Emerging applications also include the evaluation of pathologies involving the lung, spleen, brain, pancreas, bowel, kidneys, female pelvis, scrotum, and the lymphatic system [78, 79].

**Table 3:** Advanced MR imaging techniques or novel contrast media as alternatives to iodinated contrast media (ICM) and gadolinium-based contrast agent (GBCA)

Alternatives to imaging studies with ICM and GBCA	Indications and advantages	Limitations
Contrast-enhanced ultrasound (CeUS) (e.g. SonoVue, which is a blood pool agent, and sonazoid, which is a liver-specific agent)	- Reported for intravenous use to evaluate focal liver lesions, blunt abdominal trauma, lung, spleen, brain, pancreas, bowel, kidneys, female pelvis, scrotum, and the lymphatic system - Quantification capabilities for evaluation of dynamic enhancement of focal lesions - Liver-specific agent is taken up by Kupffer cells and behave like group III GBCA - Non-nephrotoxic, excreted in the lungs - Few adverse effects, majority are mild [52]	- Very rare anaphylactic reactions - Operator-dependent technique - If multiple lesions are present or the scan range is extensive, repeated doses of ultrasound contrast agent administration may be needed
Time-of-flight (TOF) MR angiogram	- TOF utilises the physical “spin” properties of proton to generate differential signal intensity between fast flowing blood and adjacent static structures - TOF MRA is best-suited for imaging intracranial vessels where longer scan times are not a problem due to lack of motion in the brain - No GBCA required, therefore no risk of nephrogenic systemic fibrosis	- Stenosis can be overestimated and artefacts can be produced by turbulent blood flow in tortuous vessels - Unable to evaluate the flow dynamics
Phase-contrast (PC) MR venogram	- Phase-contrast MR utilises the physical properties of “moving spin”. By measuring the changes in phases in moving spins, the flow-related velocity can be calculated and encoded - Best suited for imaging slow flow intracranial vessels where longer scan times are not a problem due to lack of motion in the brain - No GBCA required, therefore no risk of nephrogenic systemic fibrosis	- Longer acquisition time than TOF MR angiogram, therefore motion artefacts can occur - Unable to evaluate the flow dynamics
Four-dimensional flow MR, or 3-dimensional time-resolved phase-contrast MRI	- A time-resolved 3D velocity field that is created by the encoding of velocity through the cardiac cycle along all three spatial dimensions, enabling the recording of three-directional velocity measurements with the use of interleaved four-point velocity encoding - Comprehensive visualisation of blood flow and haemodynamics, especially useful in cardiovascular imaging - GBCA can be used to augment scan quality but is not often necessary, therefore minimal to no risk of nephrogenic systemic fibrosis	- Motion artefacts due to physiological movements such as cardiac and respiratory motions, resulting in suboptimal scan quality. Gating to such motions can be challenging - Relatively long scan time
Arterial spin labelling (ASL) MR perfusion study	- ASL magnetises the protons in the inflowing arterial blood using radiofrequency pulses. By subtracting the non-labelled control MR image with the labelled image, perfusion-weighted imaging can be generated - Most commonly used for evaluation of cerebral blood flow or perfusion of focal intracranial lesion; renal perfusion has also been studied - No GBCA required, therefore no risk of nephrogenic systemic fibrosis	- Long scan time and subjected to motion artefacts
Ferumoxytol-enhanced MRI	- Iron-based contrast agent, therefore no risk of nephrogenic systemic fibrosis - Intravenous ferumoxytol has a prolonged blood pool phase, allowing high-resolution vascular and vessel wall imaging - Delayed intracellular uptake produces GBCA-like enhancement pattern on T1W sequences around 24 h after administration	- Rare anaphylactic reaction - Slow clearance, which may produce confounding results if short interval repeat ferumoxytol-enhanced MR is needed - Contraindicated in patients with haemochromatosis or haemosiderosis
Carbon dioxide (CO_2_) angiography	- Alternative to ICM and behaves as negative contrast - Non-nephrotoxic, excreted in the lungs - Virtually no limit to the number of runs or amounts of CO_2_ used - Safety of CO_2_ over other gases is attributed to its much higher tissue solubility, virtually eliminating the risk of serious complications from inadvertent gas embolism	- Air contamination: Unlike liquid contrast agents, CO_2_ is colourless and cannot be distinguished from air. Hence, contamination with air due to breached technique or equipment failure can be unrecognised - Cannot be used in supradiaphragmatic and cerebral angiography due to risk of cerebral air embolism [80, 81]

UCAs are microbubbles composed of an inert gas encapsulated by a phospholipid and/or protein shell. The gas component is eliminated via exhalation, while the shell undergoes hepatobiliary excretion [82]. Because UCAs are not excreted by the kidneys, contrast-enhanced ultrasound (CEUS) can be safely performed in children with impaired kidney function, those receiving potentially nephrotoxic medications, and neonates with immature renal physiology. UCAs have an excellent safety profile and, unlike GBCAs, do not exhibit soft tissue deposition. CEUS is also advantageous in critically ill patients, as it can be performed at the bedside, avoiding the need for transport. This portability facilitates continuous patient monitoring and helps maintain thermal stability in vulnerable neonatal populations.

**Fig. 4:**
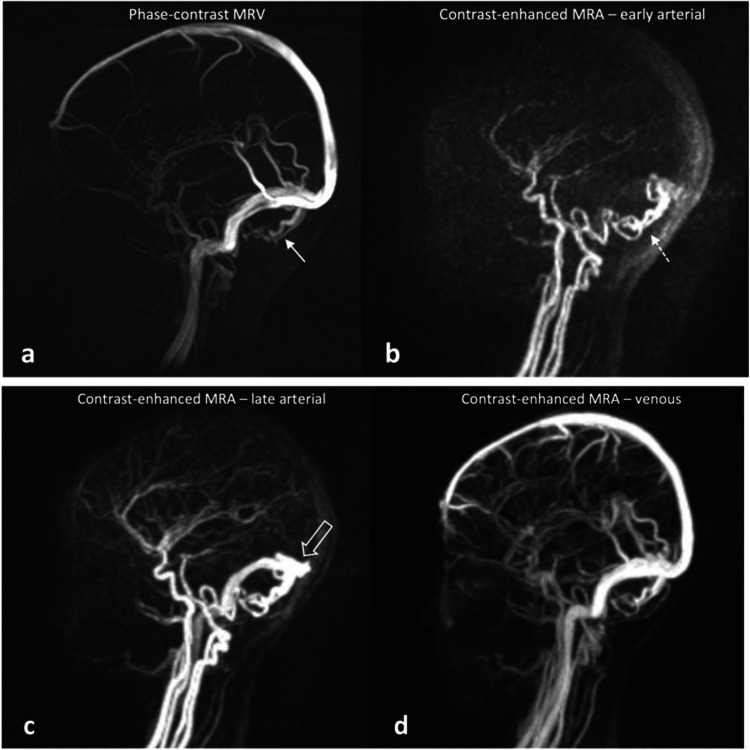
Phase-contrast MR venogram (**a**) and contrast-enhanced dynamic MR angiogram (**b**–**d**) acquired in a 9-year-old boy with capillary malformation–arteriovenous malformation (CM-AVM) syndrome. **a** Phase-contrast MR venogram shows some dilated vascular structures at the posterior fossa (white arrow); however, without dynamic contrast-enhanced sequences, it was unable to differentiate whether these structures represent dilated arteries or veins. **b** The early arterial phase of contrast-enhanced dynamic MR angiogram confirmed that these are dilated posterior inferior cerebellar arteries. **c** Early opacification of the transverse sinus (open white arrow) observed on the late arterial phase MR angiogram confirmed the presence of cerebellar pial arteriovenous fistula. **d** The rest of the dural venous sinuses were opacified at the appropriate timepoint in the venous phase of the MR angiogram

Magnetic resonance (MR) vascular imaging and perfusion studies can be performed without GBCAs by leveraging the intrinsic physical properties of proton relaxation times (Fig. 4). Advanced GBCA-free techniques include time of flight (TOF), phase contrast, and arterial spin labelling (ASL), each offering distinct advantages and limitations that are beyond the scope of this article. In addition, ferumoxytol, a non-nephrotoxic iron oxide nanoparticle, has recently emerged as an alternative to GBCAs, particularly in patients with severe kidney impairment. Its prolonged intravascular circulatory time and unique pharmacokinetic profile have demonstrated potential in vascular, oncologic, and neurologic MR imaging applications [83]. Artificial intelligence has also rendered contrast-free MR a possibility in cardiac imaging. A recent study highlighted the use of virtual native enhancement to produce a late-gadolinium-like image without the need of GBCA for evaluation of myocardial scarring, with a specificity of 100% and a sensitivity of 77% [84].

Carbon dioxide (CO_2_) angiography is a non-nephrotoxic alternative to conventional angiography that uses ICM. Due to its high solubility and rapid pulmonary elimination, CO_2_ can be safely injected into arteries below the diaphragm and into the venous system without causing clinically significant gas embolism. As a negative contrast agent, CO_2_ is particularly useful in a range of peripheral angiographic applications in patients with CKD. In the paediatric population, its most relevant application is renal angiography, particularly in the evaluation and treatment of renal artery stenosis.

## Conclusion and the way forward

**Fig. 5:**
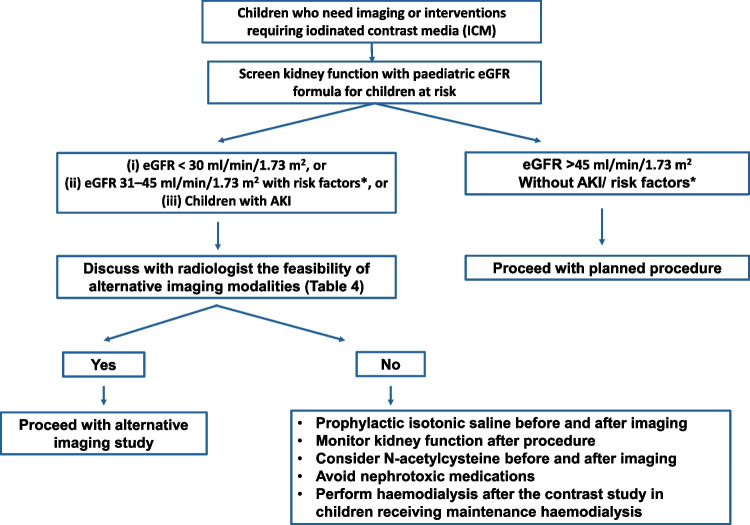
Management algorithm in children at risk of developing contrast-induced acute kidney injury due to iodinated contrast media. Abbreviations: AKI, acute kidney injury; eGFR, estimated glomerular filtration rate. *Risk factor: Pre-existing renal insufficiency, patient on dialysis, post-renal transplant, single kidney, diabetes mellitus, hypertension, heart disease, and malignancy involving the kidney

**Fig. 6:**
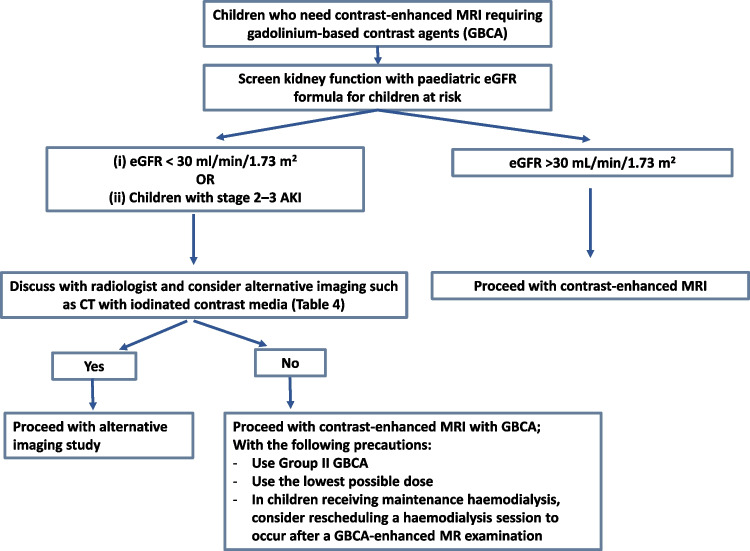
Management algorithm in children at risk of developing nephrogenic systemic fibrosis due to gadolinium-based contrast agents. Abbreviations: AKI, acute kidney injury; eGFR, estimated glomerular filtration rate; MRI, magnetic resonance imaging

ICMs and GBCAs are essential in improving diagnostic accuracy and procedural outcomes in a diverse range of radiological examinations and image-guided interventions. However, it is critical to assess the benefit–risk ratio prior to their administration, particularly in at-risk paediatric patients with pre-existing kidney impairment or AKI. In such cases, the use of alternative contrast agents or imaging strategies should be carefully considered. In situations where ICMs or GBCAs cannot be avoided, various preventive measures can be adopted to mitigate the risk of developing contrast-related adverse outcomes (Figs. 5 and 6). Multidisciplinary discussion and close collaboration between paediatric radiologists and nephrologists are crucial to determine the most appropriate imaging approach and to ensure that preventive strategies are tailored to the individual patient. Further prospective clinical trials are needed to better define the incidence of contrast-induced adverse outcomes and to establish evidence-based preventive practices in the paediatric population.

## Supplementary Information

Below is the link to the electronic supplementary material.

ESM 1 Graphical abstract (PPTX 340 KB)

## Data Availability

Data sharing not applicable to this article as no datasets were generated or analysed during the current study.
