# Supplementary material for: Contrast-induced acute kidney injury and nephrogenic systemic fibrosis in children
Source: Pediatr Nephrol. 2025 Aug 11;41(4):957–72. doi: 10.1007/s00467-025-06916-w (PMC12953423; doi:10.1007/s00467-025-06916-w)
Supplement: Supplementary file 1 — Graphical abstract (PPTX 340 KB) [file 467_2025_6916_MOESM1_ESM.pptx]

## Slide 1
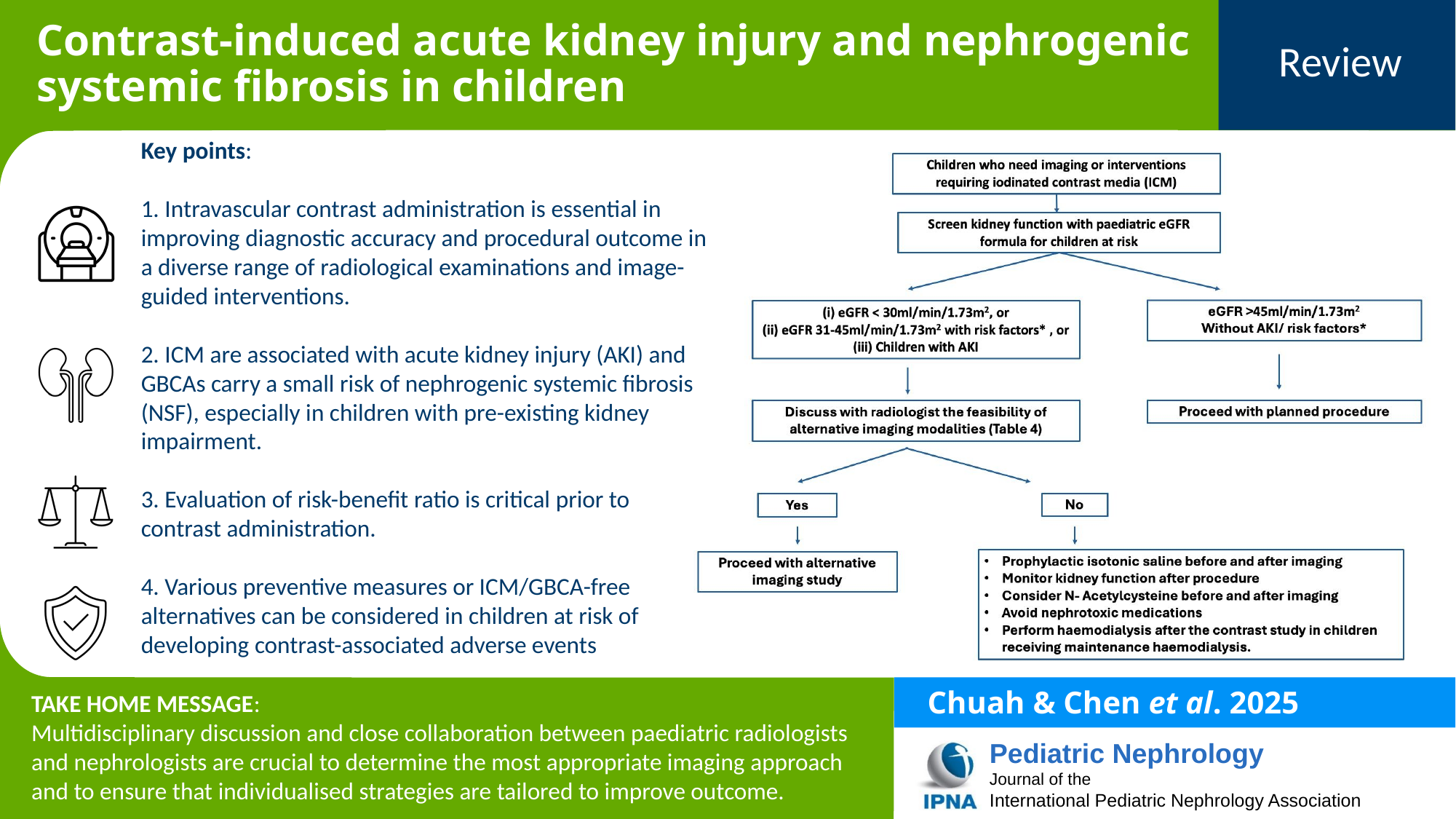

Contrast-induced acute kidney injury and nephrogenic systemic fibrosis in children
Key points:
1. Intravascular contrast administration is essential in improving diagnostic accuracy and procedural outcome in a diverse range of radiological examinations and image-guided interventions.
2. ICM are associated with acute kidney injury (AKI) and GBCAs carry a small risk of nephrogenic systemic fibrosis (NSF), especially in children with pre-existing kidney impairment.
3. Evaluation of risk-benefit ratio is critical prior to contrast administration.
4. Various preventive measures or ICM/GBCA-free alternatives can be considered in children at risk of developing contrast-associated adverse events
Chuah & Chen et al. 2025
TAKE HOME MESSAGE:
Multidisciplinary discussion and close collaboration between paediatric radiologists and nephrologists are crucial to determine the most appropriate imaging approach and to ensure that individualised strategies are tailored to improve outcome.
